# Supplementary material for: Real-time Hall-effect detection of current-induced magnetization dynamics in ferrimagnets
Source: Nat Commun. 2021 Jan 28;12:656. doi: 10.1038/s41467-021-20968-0 (PMC7843968; doi:10.1038/s41467-021-20968-0)
Supplement: Supplementary file 1 — Supplementary Information [file 41467_2021_20968_MOESM1_ESM.pdf]

Real-time Hall-effect detection of current-induced magnetization dynamics in ferrimagnets

G. Sala[1*], V. Krizakova[1], E. Grimaldi[1], C.-H. Lambert[1], T. Devolder[2], and P. Gambardella[1*]

[1]*Department of Materials, ETH Zurich, 8093 Zürich, Switzerland*
[2]*Centre de Nanosciences et de Nanotechnologies, CNRS, Universitè Paris-Sud, Universitè Paris-Saclay, 91405 Orsay Cedex, France*

Table of contents

Note 1. Sensitivity of the technique

Note 2. Temporal resolution of the technique

Note 3. Sample characterization

Note 4. Measurement protocol and analysis of raw signals

Note 5. Compensation of resistance offsets

Note 6. Switching with short pulses

Supplementary References

Supplementary Note 1. Sensitivity of the technique

The smallest detectable signal is determined by the relative amplitude of the time-resolved anomalous Hall signal and the noise of the electric circuit. In what follows, we estimate the sensitivity of our technique by calculating the anomalous Hall voltage generated by the magnetic dots (see Fig. S1a), the time-resolved amplified voltage, and the superimposed noise.

The anomalous Hall voltage V_H depends on the transverse anomalous Hall resistance R_{xy} and on the current I_x, thus it can expressed as

$$V_H = R_{xy} I_x = R_{xy} \frac{V_P}{R_I},$$

where V_P is twice the amplitude $V_P/2$ of the positive (or negative) pulse in Fig. S1b, and R_I the resistance of the injection line. R_{xy} is directly proportional to the anomalous Hall resistivity ρ_{xy}, but the comparison with values reported in the literature is not immediate because of geometrical reasons. First, the current distribution is highly inhomogeneous in the Hall cross. Second, most of the current flows through the Pt layer but a small portion enters also the GdFeCo dot and propagates vertically. Third, the anomalous Hall effect does not extend over the entire cross but is limited to the dot area. This geometry is very different from the typical experimental configuration used to measure ρ_{xy}, namely a multilayer Hall bar, where the current spreads out in the magnetic layer, which is continuous and extends to the transverse probes, i.e., the sensing line. To account for these differences, we introduce three geometrical parameters. We define the filling factor $F = \pi(\frac{D}{2w})^2$ as the ratio between the areas of the dot and the central portion of the cross (see Fig. S1a), arguing that the Hall signal scales with the magnetic area. In addition, we introduce the sensitivity factor ε, which represents the finite sensitivity of the probes to

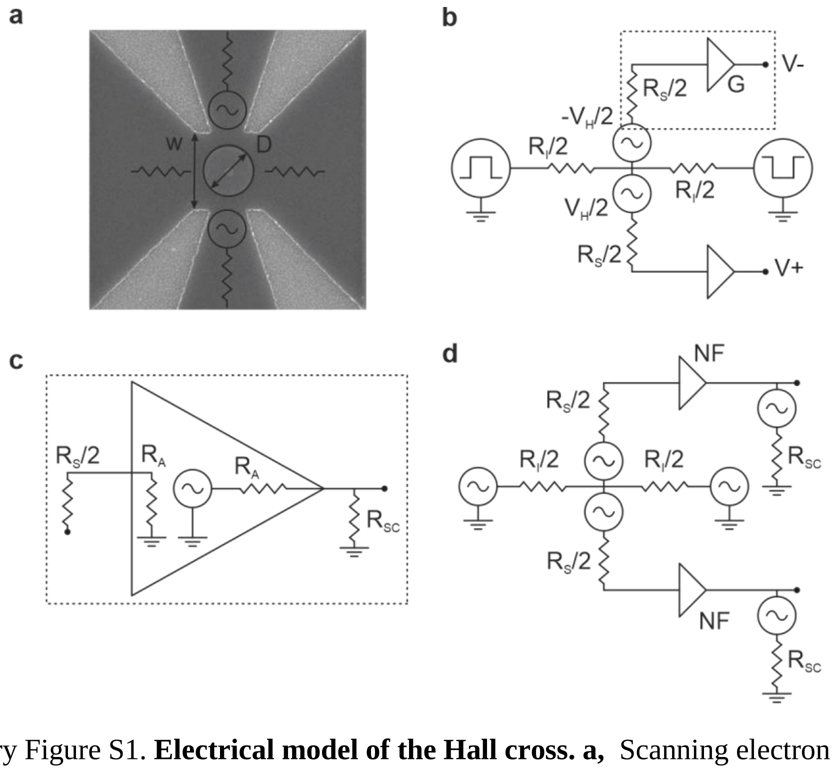

Supplementary Figure S1. **Electrical model of the Hall cross. a,** Scanning electron micrograph of the 1-µm-wide dot and Hall cross, and associated resistors. w and D are the width of the Hall cross and the diameter of the dot, respectively. **b,** Equivalent electric circuit of the Hall cross, with the associated resistors, voltage sources, and amplifier. The dashed rectangle corresponds to the schematic in **c**, which represents the equivalent model of the amplifier. **d,** Model of the Johnson noise: every resistor is replaced by an ideal noise-free resistor and a noise voltage source.

variations of the electric potential in the cross[1,2]. ε is determined by the dimension of the Hall cross and by the position of the dot with respect to its center. Finally, we add a parameter δ that takes into account the non-uniform contribution to the anomalous Hall voltage across the thickness of GdFeCo. The value of δ is determined by the specific current distribution within the volume of the dot and by the relative weight of volume and interface as sources of the anomalous Hall voltage. Therefore, given the thickness t of the Pt layer, the anomalous Hall voltage reads

$$V_H = \varepsilon\delta F \frac{\rho_{xy}}{t}\frac{V_P}{R_I}. \quad (1)$$

This voltage is then amplified and measured in real time. Figure S1b presents the equivalent electric circuit of the Hall cross. We model the device with four resistors ($2 \times \frac{R_I}{2}, 2 \times \frac{R_S}{2}$) connected through a central node. The resistors represent the two branches of the injection line with total resistance R_I, along which the pulses are injected, and the two branches of the sensing line with resistance R_S used to probe the anomalous Hall voltage. Since the resistance difference between the two branches of the sensing (injection) line is a few Ohm at most, we assume for simplicity that the branches are equal in pairs. For the sensing line, this hypothesis is equivalent to considering an ideal offset-free transverse voltage. The anomalous Hall effect can be modelled by two voltage supplies of opposite sign ($\pm\frac{V_H}{2}$) placed along the two sensing branches. At the centre of the cross, the counter-propagating pulses enforce a virtual ground. Then, the differential Hall signal S measured at the input ports of the oscilloscope is the result of the amplified voltage partition between $\frac{R_S}{2}$ and the input resistance of the amplifier R_A:

$$S = 2G\frac{V_H}{2}\frac{R_A}{R_A + \frac{R_S}{2}} \quad (2)$$

Here, the amplifier is treated as a simple ideal amplifying stage with gain G and $R_A = 50$ Ohm input and output impedances (see Fig. S1c). The input resistance of the oscilloscope is also $R_S = 50$ Ohm.
The measured amplified signal is accompanied by noise, which originates mainly from the Johnson noise of the resistors (N_J), the noise of the pulse generator (N_P) caused by its output impedance, the resolution of the oscilloscope N_{SC}, and, above all, the noise figure (NF) of the amplifiers. Additional noise sources are the passive electric devices present in the circuit (bias-Tees, couplers, balun divider). Moreover, the wire bonds and our printed circuit board pick up electromagnetic disturbances from the environment. However, these extra noise contributions are negligible compared to N_J, N_P, N_{SC}, and NF.
We model the Johnson noise by replacing each resistor in Fig. S1b with the equivalent Thevenin circuit, comprising of an ideal resistor of the same resistance R and voltage source $N_J = \sqrt{2}\,N_{rms} = \sqrt{8k_BT\Delta f R}$, with $k_BT \approx 4.1 \times 10^{-21}$J the thermal energy and $\Delta f \approx 50$ MHz the bandwidth (20 ns pulses). The resulting equivalent noisy circuit is sketched in Fig. S1d. It can be simplified by condensing the contributions of all the resistors into a single effective resistance R_{eff}:

$$R_{eff} = \left(\frac{\frac{2R_AR_I}{2R_A + R_S + 2R_I}}{\frac{R_I}{2} + \frac{(R_A + \frac{R_S}{2})R_I}{2R_A + R_S + 2R_I}}\right)^2 \frac{R_I}{2} + \left(\frac{R_A + \frac{2R_AR_I}{8R_A + 4R_S + 2R_I}}{R_A + \frac{R_S}{2} + \frac{(R_A + \frac{R_S}{2})R_I}{4R_A + 2R_S + R_I}}\right)^2 \frac{R_S}{2}.$$

Then, the input noise to each amplifier is

$$N_{in} = \sqrt{8k_BT\Delta f R_{eff}} + \frac{R_A}{R_A + \frac{R_S}{2}}N_P.$$

Considering also the Johnson noise of the oscilloscope's input impedance R_{SC} and the digital-to-analogue quantization, the total noise superimposed to the signal reads

$$N = 2\left(GN_{in} + 10^{\frac{NF}{10}}GN_{in} + \sqrt{8k_BT\Delta fR_{SC}} + \frac{10V_R}{2^8}\right), \quad (3)$$

where the first term represents the amplified sum of the Johnson and pulse generator noises, the second term the noise introduced by the amplifier, and the third term the Johnson noise of the input impedance R_{SC} of the oscilloscope. Strictly speaking, the last contribution in Eq. (3) is not noise, but the intrinsic finite sensitivity of the oscilloscope. This resolution is determined by the number of bits (8) and divisions (10), and the voltage range (V_R). Finally, the factor 2 is due to the mathematical subtraction of the amplified V_+ and V_-.

Equations (1)-(3) can be used to estimate the signal-to-noise ratio (S/N) and the sensitivity of the setup. In our case: D = 1000 nm, w = 1500 nm, R_I = 360 Ohm, R_S = 806 Ohm, R_{SC} = 50 Ohm, R_A = 50 Ohm, G = 20 (26 dB), NF = 6 dB, N_P = 9 µV, V_R = 7 mV, ε = 0.4 (Ref. [2]). These values lead to: R_{eff} = 16 Ohm, N_{in} = 6.1 µV, F = 0.35. We assume a pulse with amplitude V_P = 2.2 V, an anomalous Hall resistivity ρ_{xy} = 10 µOhm cm (Refs. [3,4]), and δ = 0.21, the latter being chosen to match the experimental R_{xy} = 0.6 Ohm. Thus, we obtain V_H = 3.6 mV and S = 7.9 mV. This last result is in good agreement with the experimentally measured value (cf. Fig. 2a). The total noise amounts to N = 1.8 mV. This value is to a large extent (up to 54%) determined by the noise figure of the amplifier, which intensifies the Johnson noise N_{in} of the circuits. This noise is expected to become more severe as the pulse length is reduced, i.e., the bandwidth is enlarged. For a 1-ns long pulse, it may increase by 4-5 times. The second largest contribution (30%) originates from the signal quantization, whereas the contribution of R_{SC} is negligible. On the basis of these figures, we estimate that the signal-to-noise ratio of a single measurement is of the order of $S/N \approx 4.4$. Since the time traces are obtained by subtraction of two measurements (see Supplementary Note 4), the S/N of the individual time trace (single-shot measurements) reduces to ≈ 2.2. By averaging over 1000 switching traces, the ratio can be improved by a factor of 30, which gives $S/N \approx 66$. This estimate matches reasonably well the actual signal-to-noise ratio of the average traces in Fig. 2a (bottom panel, 2.2 V pulse amplitude), which have about 6.5 mV and 0.15 mV signal and root-mean-square noise amplitude, respectively.

These considerations explain why our technique is advantageous. Without the compensation of the pulses at the centre of the Hall cross, the transverse signals V_+ and V_- are of the same order of magnitude as the injected pulse, e.g., 1 V. The magnetic signal is thus a tiny variation on the order of a few mV on top of the large background. In such conditions, a much higher range V_R is required to accommodate the entire signal into the available divisions of the oscilloscope. As a consequence, the finite vertical resolution becomes dominant over the rest of the noise and masks the magnetic signal. Sourcing the oscilloscope with the differential signal $V_+ - V_-$ would definitely improve the resolution by removing part of the background. Still, this approach would not solve completely the problem, because of the asymmetries between the sensing branches. In contrast, our technique minimizes the current spread and hence allows for exploiting the full acquisition range of the oscilloscope to probe only the magnetic signal.

This analysis suggests also a few directions for further improvements. In the first place, the device geometry and the materials (thickness, resistivity) could be designed to maximize V_H. For example, the anomalous Hall resistance could be enhanced by increasing the ratio between the width of the sensing arms and the dot diameter[5,6], so as to increase the factor ε. Likewise, the central area of the cross should be made the smallest possible, compatibly with the dot size. This optimization becomes fundamental when downscaling the devices to sub-µm dimensions. However, the device optimization is not free from constraints because the anomalous Hall voltage, the current density required to induce the magnetization switching, the geometry of the Hall cross, and its resistance are not independent. For instance, the device

miniaturization, which would enlarge V_H, would also increase the resistance of both the injection and sensing lines, hence the Johnson noise. Therefore, an alternative option is the optimization of the setup. At the present stage, the critical source of noise in our circuitry is the voltage amplifier. With all other parameters fixed, amplifiers with a 1 dB noise figure are expected to provide S/N = 3.5 for the single-shot traces. Additionally, the subtraction of V_+ and V_- prior to detection by the oscilloscope should improve the S/N by permitting the reduction of V_R. If V_R is reduced to the minimum of our oscilloscope (2 mV), then the S/N would further increase to 5.4. The subtraction could be done with an additional balun used in the opposite configuration, namely, with the input signals V_+ and V_- connected to the inverting and non-inverting ports of the device.

Supplementary Note 2. Temporal resolution of the technique

As described in the main text, the temporal resolution is determined by the sampling and by the acquisition mode (real time, interpolated real time, etc.). In this work, the traces were acquired in the interpolated real-time mode, which allows for a nominal temporal resolution of ≈ 100 ps, sufficient to track the dynamics of ns-long pulses. For shorter pulses, the nominal resolution could be improved to a few ps by using a faster oscilloscope. We note that the other elements of the circuit and the cabling may distort the shape of the electrical excitation if their transfer function does not match the required frequency range, but they do not influence the temporal resolution. Instead, it is of primary importance to ensure the equal length and symmetry of the injection (sensing) lines of the circuits to guarantee the synchronization of the injected (sensed) signals.

The shortest traces that we could reliably measure correspond to 2-3 ns-long pulses. This limitation has a different "extrinsic" origin than the circuit components, namely the geometry of the Hall cross, which was not specifically designed for transmitting rf pulses, and, above all, the use of wire bonds to contact the device, which are inductively coupled. As a consequence, the raw traces have edge spikes with about 1 ns FWHM (see Fig. S4c) that complicate the analysis of the magnetic traces for pulses shorter than 1 ns. The replacement of the wire bonds with rf probes would improve the transmission of sub-ns pulses. We stress that these limitations affect the length of the pulses, but not the temporal resolution, which remains 100 ps and can be independently improved.

Supplementary Note 3. Sample characterization

Figure S2a reports the hysteresis loops of a $Gd_{30}Fe_{63}Co_7$ device as probed by static measurements of the anomalous Hall resistance, with field applied perpendicular to the plane (polar angle = 0°) and almost in plane (89°). The sense of rotation of the hysteresis loop indicates that the magnetization is dominated by the transition metals Fe and Co. The GdFeCo layer has perpendicular magnetic anisotropy, with an effective anisotropy field of the order of 300 mT. The saturation magnetization was estimated to be 25 kA/m using SQUID magnetometry performed on a full film sample. The device can be reliably switched between the up and down states by bipolar electric pulses in presence of an in-plane magnetic field collinear with the current direction, as typical of spin-orbit torques (see Fig. S2b).

We note that the GdFeCo devices studied here belong to a batch of samples with variable Gd concentration that cross the magnetization compensation temperature. However, we found that the fabrication steps alter the properties of the devices with respect to those of the full films. This undesired change is one of the limitations of amorphous ferrimagnets, which are particularly sensitive to standard operations such as the ion milling and the resist baking. These issues have already been observed by other groups (see e.g., Ref. [7–9]) and are possibly caused by the selective oxidation or migration of the rare-earth atoms[10]. Our estimate, based on the variation of the magnetization compensation temperature with the Gd concentration (about 30 K every 1%), is that the magnetization compensation temperature is around 250 K. Because of Joule heating during pulsing, we are confident that the magnetization of

our devices is always “FeCo-like” for the time-resolved Hall effect experiments reported in this work, which were all performed in ambient conditions.

In order to determine the working point required to induce the switching, we measured the probability of switching as a function of in-plane magnetic field and pulse amplitude. To this aim, we used the dc sub-network of the circuit shown in Fig. 1 in the main text. The procedure was the following. We applied a sequence of set-reset rf pulses with identical length and amplitude but opposite polarity. The variation of the transverse dc resistance before and after each pulse was compared with the anomalous Hall resistance to assess the outcome of the pulse: if the variation was larger than 75 % of this reference, we considered that the pulse succeeded in switching the magnetization. Every pulse sequence comprised 50 set-reset pairs of pulses, and the switching probability was defined by the ratio of successful pulses to 50. We repeated this procedure for different pulse lengths, amplitudes, and fields, as reported in Fig. S3a-d. As expected, the minimum voltage for 100% switching decreases as the field or the pulse length are increased.

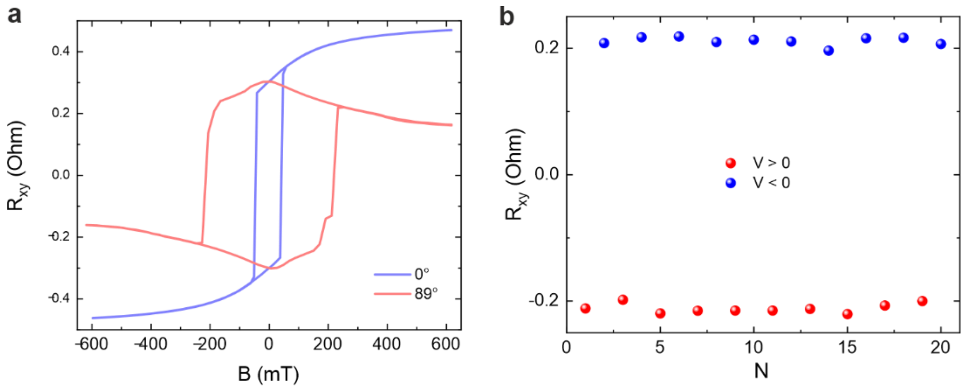

Supplementary Figure S2. **Sample characterization. a,** Hysteresis loops measured by the anomalous Hall resistance with the field applied out of plane (0°) and in plane (89°). **b,** Switching of the magnetization by a sequence of positive set (V > 0) and negative reset (V < 0) pulses with length of 20 ns and amplitude of 1.6 V. The in-plane field was 150 mT. Note that the switching amplitude is smaller than the anomalous Hall amplitude in **a** because of the tilt induced by the applied field (cf. with the red trace in **a** at 150 mT).

Supplementary Note 4. Measurement protocol and analysis of raw signals

In an ideal scenario, the signal measured by the oscilloscope should approximately resemble a “rectangle”, that is, it should replicate the temporal profile of the applied electric pulse. In such a case, the amplitude of the signal (height of the rectangle) would already represent the measurement of the magnetization state. If the magnetization was in equilibrium, the amplitude would remain constant, to a high or low level in dependence of the up or down orientation of the magnetization. During the switching, instead, the trace would transition from one level to the other. However, spurious nonmagnetic contributions alter the ideally-rectangular profile of the measured signal. These contributions have multiple origins. First, the edge of the pulses have large spikes caused by the inductive coupling between the wire bonds and the electric contacts of the PCB. Second, the device itself, which is not adapted to radio frequencies, distorts the pulses and hence the measured signal. In

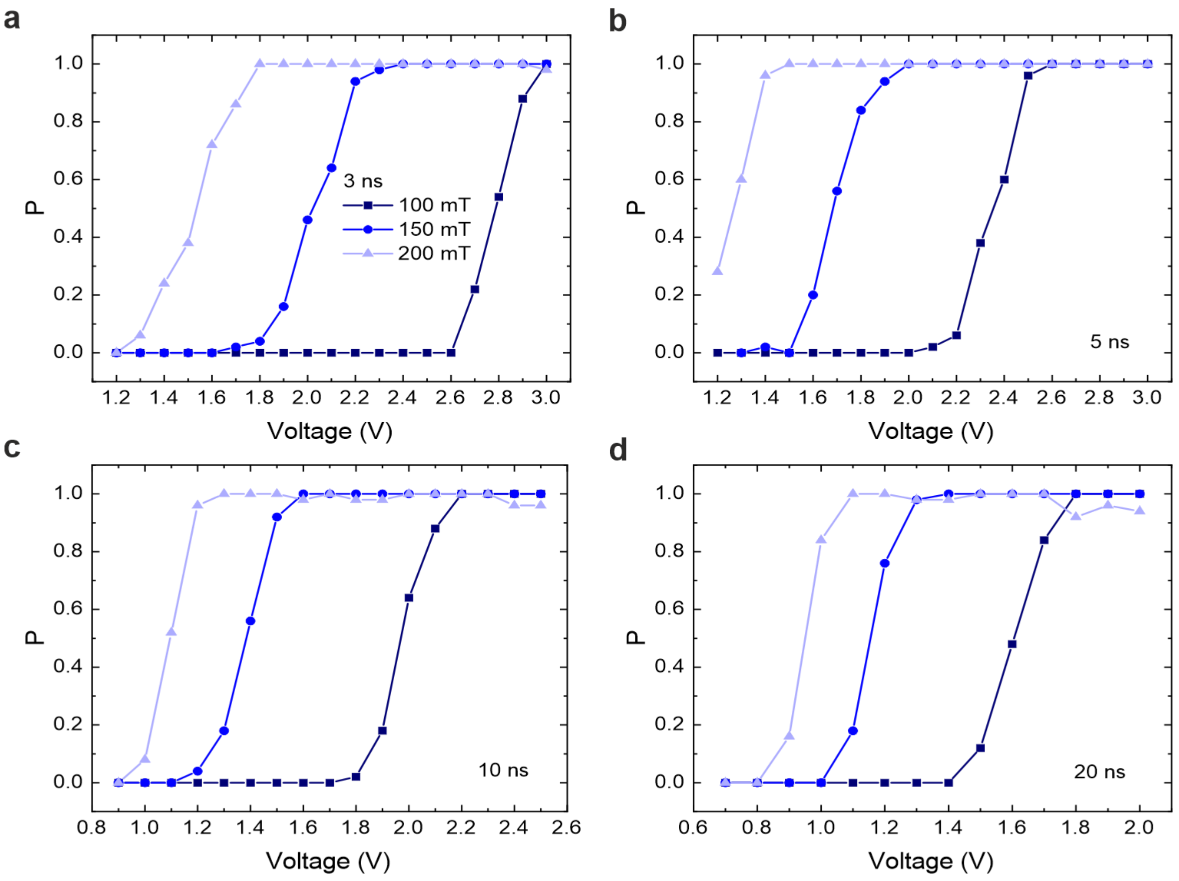

Supplementary Figure S3. **Switching probability. a-d,** After-pulse switching probability as a function of pulse amplitude, for different pulse lengths and in-plane fields.

addition, the voltage amplifier introduces high-frequency oscillations. Finally, as shown in Fig. S4a,b, the voltage difference between the inverted (I) and non-inverted (NI) pulses at the output of the balun divider is several mV, which is about 1% of the pulse amplitude. This component is quite small with respect to the input pulse. Yet, the unbalance causes a residual current leakage through the transverse arms which adds a small voltage offset (comparable to or smaller than the magnetic signal). Therefore, the magnetic signal is better extracted from the raw traces by comparing measurements of a reference and the switching and removing the non-magnetic part. In fact, every trace of the same type as in Fig. 2b-e of the main text results from the combination of two measurements. The procedure that we adopt to isolate the magnetic signal is the following[11].

First, in the presence of a positive in-plane magnetic field, we acquire a background signal by repeatedly injecting identical pulses with the same current direction and amplitude. In these conditions, the magnetization remains in the equilibrium state, which is determined by the field direction and the sign of the spin-orbit torques defined by the current polarity. The latter equals the polarity of the pulse travelling along $+x$, that is, from left to right in Fig. 1a in the main text. The differential voltage S measured during each pulse is nominally always the same, but we average over multiple pulses, typically 5000, to reduce the noise. Then, we repeat the same step for the opposite field direction and the same current polarity, to acquire the background signal corresponding to the opposite equilibrium state (see Fig. S4c). By definition, all the undesired contributions do not change with the magnetic configuration of the device, hence they can be removed by subtracting the two signals. Their difference yields the net magnetic contrast: reference trace = Background ($B < 0$) – Background ($B > 0$). This is the black trace shown in Fig. S4d as well as in Fig. 2a. Since for $V > 0$ and $B < 0$ ($B > 0$), the magnetization remains in

the up (down) state, corresponding to positive (negative) anomalous Hall voltage, the reference trace so defined has positive sign.

Next, we acquire the signal corresponding to the switching of the magnetization by slightly varying the procedure, that is, by delivering a train of set-reset pulses with alternating polarity. Now, at each pulse the current direction changes and so does the magnetization. For example, for positive field, the positive current causes the up-down switching, whereas the successive negative current induces the down-up reversal. By averaging over 1000 pulses of the same polarity, we acquire the green signal shown in Fig. S4c (a positive in-plane field is applied). It coincides initially with the signal for Background ($B < 0$) (magnetization up) and during the pulse it transitions to the signal for Background ($B > 0$) (magnetization down). Therefore, similarly to the reference trace, the signal S associated to a switching event is combined with one of the two backgrounds: switching trace = S ($B > 0$) – Background ($B > 0$). The application of this procedure leads to the blue trace in Fig. S4d as well as to the traces in Fig. 2. The ≈ 0 mV (≈ 5 mV) trace level identifies the uniformly-magnetized down (up) state, whereas any deviation of the traces from the top and bottom levels correspond to a tilt of the magnetic moments or to a multi-domain configuration. Finally, the normalization of the switching trace to the reference trace provides the purely-magnetic time traces (cf. Fig. 2b-e in the main text). The same identical approach is used for detecting single-shot events, with the only difference that, instead of averaging, every single switching signal is recorded. The procedure that we adopt to measure and remove the background signal is very similar to that reported in Ref. [12]. Therefore, our measurement protocol is comparable to that of standard time-resolved Hall measurements.

Finally, we note that the reference trace can be acquired by using protocols different from ours, which is adapted to the specific case of spin-orbit torque switching. For example, the background signals of

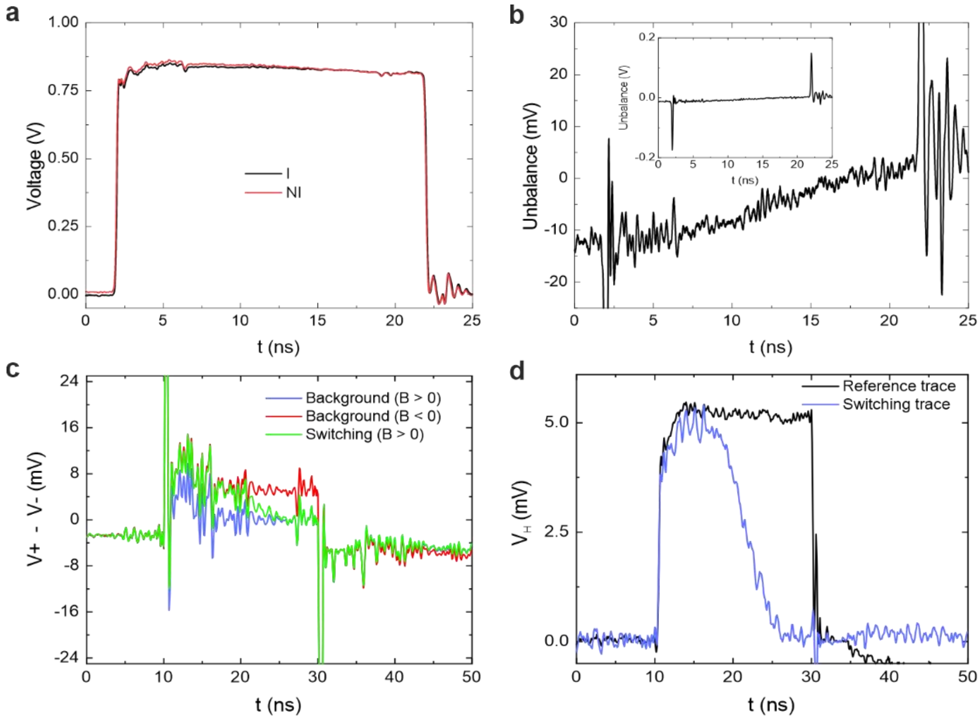

Supplementary Figure S4. **Analysis of raw signals. a,** Inverted (I) and non-inverted (NI) pulses at the outputs of the balun divider, for a 20 ns, 1.6 V input pulse; the sign of the I pulse has been inverted for comparison. **b,** Close-up of the difference between the I and NI pulses (I - NI). Inset: full voltage difference between the two pulses. **c,** Average raw electric signals corresponding to the background, for the two in-plane field directions, and to the switching (for positive field). **d,** Reference and switching traces obtained by subtraction of the signals in **c**.

perpendicularly-magnetized samples could also be acquired by fixing the magnetization with out-of-plane fields. If the polarity of the current has an effect, a reference could also be obtained by comparing background signals measured with opposite current polarity. Alternatively, the signal measured with a low-amplitude pulse could be used as background: under the assumption that the low amplitude does not produce magnetic changes, the corresponding trace could be subtracted from a higher-amplitude trace after proper rescaling. In antiferromagnets, repeated pulses produce a memristive-like switching. Then, the background trace could be obtained after applying a sequence of repeated pulses that saturate the read-out signal to the maximum (or minimum) level. Therefore, in general, the measurement protocol can be adapted to the specific application.

Supplementary Note 5. Compensation of resistance offsets.

Our technique does not imply a more complex circuit or measurement protocol than traditional differential Hall measurements. For comparison, we consider the work by Yoshimura et al. (Ref. [12]). In our setup, we included DC components to simultaneously access the static electric and magnetic properties of the devices. Once the DC subnetwork, which is not necessary for time-resolved measurements, is removed, the sole difference between the differential Hall measurement presented in Ref. [12] and our technique is the balun divider. The balun is a simple, small, and affordable component that does not require any power supply and easily fits into any electrical setup.

As an additional advantage, our technique allows for compensating possible resistive offsets that are caused by the imperfect fabrication or are intrinsic to asymmetric devices. To prove this point, we have measured the raw electrical signals corresponding to the “up” and “down” magnetization states in a Hall bar device with two off-centered Hall crosses (see Fig. S5a). In contrast to the symmetric Hall cross considered in the manuscript, in this device the electric potentials determined by the two pulses at the center of the right Hall cross are different because of the asymmetric resistance load. As a result, the current does flow in the transverse arms and the signals measured on the oscilloscope present a finite offset (see Fig. S5b). Since this offset is not negligible, to acquire the signals we could not use the the maximum vertical resolution of the oscilloscope. Such problems can be circumvented by correcting the pulses amplitudes to enforce the virtual ground at the position of the Hall cross. In the specific case discussed here, we added a 4 dB attenuator along the direction of the negative pulse. Thanks to this adjustment, the vertical offset was removed from the raw signal, which allowed us to exploit the highest vertical resolution of the oscilloscope. Therefore, our technique does not require the device under test to be longitudinally symmetric. Although we do not have at our disposal devices with asymmetric transverse Hall arms, we believe that transverse resistance offsets could be compensated in the same way as for the longitudinal offset. Since commercial attenuators provide attenuation steps as small as 0.5 dB (= 0.944), the amplitude of the pulses can be tuned with rather large precision. This capability is a specificity of our technique, for no such countermeasures can be taken in standard differential Hall measurements.

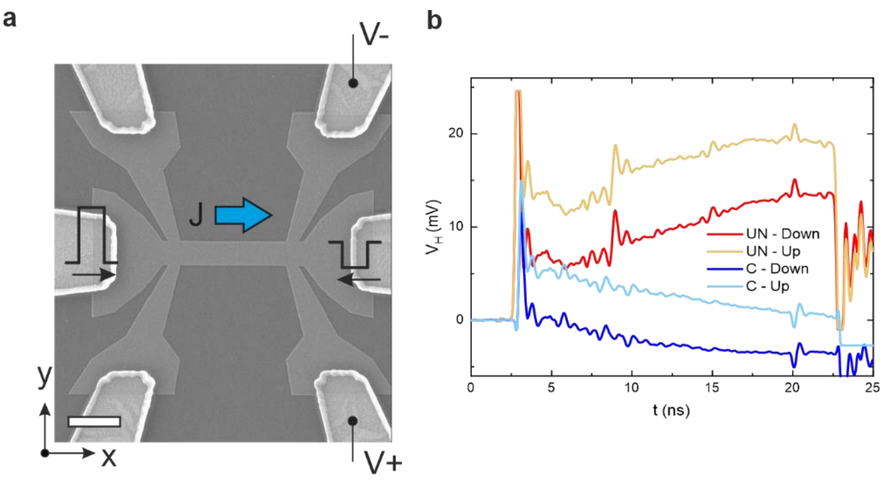

Supplementary Figure S5. **Compensation of resistance offsets. a)** Hall bar with off-centered Hall crosses. The anomalous Hall effect is measured in the right Hall cross. The negative pulse moving from right to left is attenuated by 4 dB compared to the positive pulse. The scale bar corresponds to 4 µm. **b)** Raw differential Hall voltage $V_H = V^+ - V^-$, with uncompensated (UN) and compensated (C) resistance offset, corresponding to the up and down magnetization states for current pulses that do not induce switching.

Supplementary Note 6. Switching with short pulses.

The measurements presented in the main text were performed with 20-ns-long pulses. These relatively long pulses allow us to clearly identify the different phases of the dynamics. In Fig. S6 we present additional average time-resolved measurements performed with 5-ns-long pulses. At the largest pulse amplitude the nucleation time is reduced down to about 800 ps. This decrease is consistent with the after-pulse probability measurements shown in Fig. S7a, which shows the switching probability measured as a function of the pulse amplitude and length for a constant in-plane field of 100 mT. The plot demonstrates that deterministic switching can be obtained with pulses as short as 300 ps, which implies quenching of the nucleation time at sufficiently high pulse amplitudes. From Fig. S7a we extracted the threshold switching voltage, defined as the voltage at which the device switches in 50% of the trials, and plotted it against $1/t_P$ in Fig. S7b (see also Fig. 5 in the main text). Below approximately 5 ns, the voltage increases linearly with the inverse of t_P, which is a signature of the intrinsic regime where the switching speed depends on the rate of angular momentum transfer from the current to the magnetic layer. On the other hand, the different dependence for $t_P > 5$ ns reveals the importance of thermal effects for the typical pulse lengths used in this study ($t_P = 20$ ns).

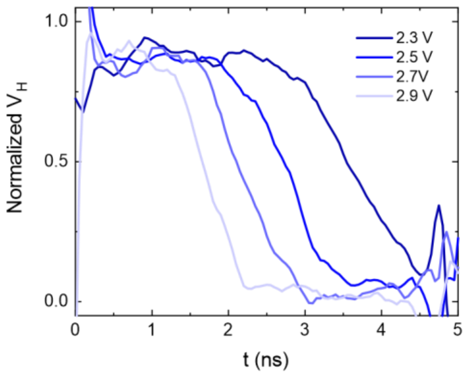

Supplementary Figure S6. **Switching with 5-ns pulses.** Normalized average traces showing the up-down magnetization switching with 5 ns-long pulses of different amplitude. Both the current and the in-plane 125 mT field were positive.

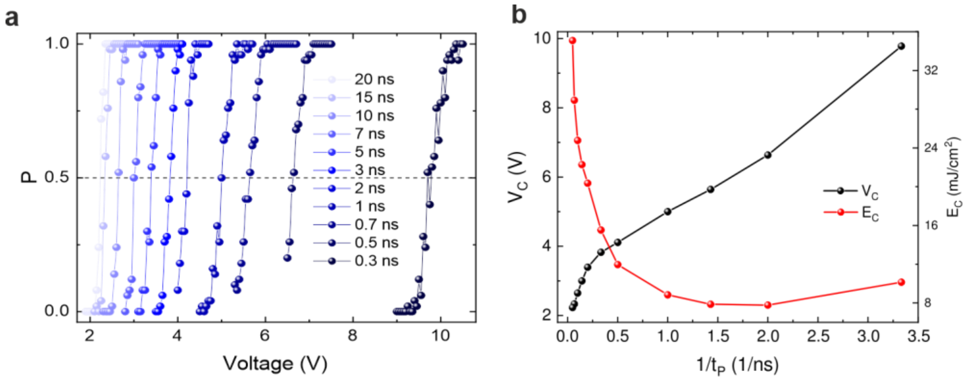

Supplementary Figure S7. **Switching as a function of pulse length. a)** Dependence of the switching probability on the pulse amplitude for different pulse lengths. Each point is the result of 50 trials. The applied in-plane field was 100 mT. Note that these measurements were performed on a different device than that used for the time-resolved measurements but they were fabricated at the same time from the same layer. **b**, Threshold switching voltage (black dots, left scale) and energy density (red dots, right scale) as a function of the inverse pulse length. The critical switching voltage is determined from **a** as the voltage at which the device switches in 50% of the trials.

Supplementary References

1. Cornelissens, Y. G. & Peeters, F. M. Response function of a Hall magnetosensor in the diffusive regime. *J. Appl. Phys.* **92**, 2006–2012 (2002).

2. Webb, B. C. & Schültz, S. Detection of the magnetization reversal of individual interacting single-domain particleswithin Co-Cr columnar thin-films. *IEEE Trans. Magn.* **24**, 3006–3008 (1988).

3. Hartmann, M. & McGuire, T. R. Relationship between Faraday Rotation and Hall Effect in Amorphous Rare-Earth—Transition-Metal Alloys. *Phys. Rev. Lett.* **51**, 1194–1197 (1983).

4. Honda, S., Nawate, M., Ohkoshi, M. & Kusuda, T. Hall effect and magnetic properties in GdFe and CoCr sputtered films. *J. Appl. Phys.* **57**, 3204–3206 (1985).

5. Kikuchi, N., Okamoto, S., Kitakami, O., Shimada, Y. & Fukamichi, K. Sensitive detection of irreversible switching in a single FePt nanosized dot. *Appl. Phys. Lett.* **82**, 4313–4315 (2003).

6. Alexandrou, M., Nutter, P. W., Delalande, M., De Vries, J., Hill, E. W., Schedin, F., Abelmann, L. & Thomson, T. Spatial sensitivity mapping of Hall crosses using patterned magnetic nanostructures. *J. Appl. Phys.* **108**, (2010).

7. Le Guyader, L., El Moussaoui, S., Buzzi, M., Chopdekar, R. V., Heyderman, L. J., Tsukamoto, A., Itoh, A., Kirilyuk, A., Rasing, T., Kimel, A. V. & Nolting, F. Demonstration of laser induced magnetization reversal in GdFeCo nanostructures. *Appl. Phys. Lett.* **101**, (2012).

8. El-Ghazaly, A., Tran, B., Ceballos, A., Lambert, C. H., Pattabi, A., Salahuddin, S., Hellman, F. & Bokor, J. Ultrafast magnetization switching in nanoscale magnetic dots. *Appl. Phys. Lett.* **114**, (2019).

9. Kirk, E., Bull, C., Finizio, S., Sepehri-Amin, H., Wintz, S., Suszka, A. K., Bingham, N. S., Warnicke, P., Hono, K., Nutter, P. W., Raabe, J., Hrkac, G., Thomson, T. & Heyderman, L. J. Anisotropy-induced spin reorientation in chemically modulated amorphous ferrimagnetic films. *Phys. Rev. Mater.* **4**, 074403 (2020).

10. Hansen, P. Magnetic amorphous alloys. *Handb. Magn. Mater.* **6**, 289 (1991).

11. Grimaldi, E., Krizakova, V., Sala, G., Yasin, F., Couet, S., Sankar Kar, G., Garello, K. & Gambardella, P. Single-shot dynamics of spin–orbit torque and spin transfer torque switching in three-terminal magnetic tunnel junctions. *Nat. Nanotechnol.* **15**, 111–117 (2020).

12. Yoshimura, Y., Kim, K., Taniguchi, T., Tono, T., Ueda, K., Hiramatsu, R., Moriyama, T., Yamada, K., Nakatani, Y. & Ono, T. Soliton-like magnetic domain wall motion induced by the interfacial Dzyaloshinskii–Moriya interaction. *Nat. Phys.* **12**, 157–161 (2016).
